# Prevalence and clinical significance of nonorgan specific antibodies in patients with autoimmune thyroiditis as predictor markers for rheumatic diseases

**DOI:** 10.1097/MD.0000000000004336

**Published:** 2016-09-23

**Authors:** Basant M. Elnady, Naglaa M. Kamal, Raneyah H.M. Shaker, Amal F. Soliman, Waleed A. Hasan, Hamed A. Alghamdi, Mohammed M. Algethami, Mohamed Bilal Jajah

**Affiliations:** a Department of Physical Medicine and Rheumatology, Benha Faculty of Medicine, Benha University, Benha, Egypt; b Department of Rheumatology, Alhada Armed Forces Hospital, Taif, Saudi Arabia; c Department of Pediatrics and Pediatric Hepatology, Faculty of Medicine, Cairo University, Cairo, Egypt; d Department of Pediatrics, Alhada Armed Forces Hospital, Taif, Saudi Arabia; e Department of Public Health, Benha Faculty of Medicine, Benha University, Benha, Egypt; f Director of Armed Forces Hospitals, Taif, Saudi Arabia; g Departments of Pulmonology and Endocrinology, Al Hada Armed Forces Hospital, Taif, Saudi Arabia.

**Keywords:** antidouble-stranded DNA, antiextractable-nuclear antigens, antinuclear antibodies, autoimmune thyroid diseases, rheumatoid factor, anticyclic-citrullinated peptides

## Abstract

Autoimmune diseases are considered the 3rd leading cause of morbidity and mortality in the industrialized countries. Autoimmune thyroid diseases (ATDs) are associated with high prevalence of nonorgan-specific autoantibodies, such as antinuclear antibodies (ANA), antidouble-stranded deoxyribonucleic acid (anti-dsDNA), antiextractable-nuclear antigens (anti-ENAs), rheumatoid factor (RF), and anticyclic-citrullinated peptides (anti-CCP) whose clinical significance is unknown.

We aimed to assess the prevalence of various nonorgan-specific autoantibodies in patients with ATD, and to investigate the possible association between these autoantibodies and occurrence of rheumatic diseases and, if these autoantibodies could be considered as predictor markers for autoimmune rheumatic diseases in the future.

This study had 2 phases: phase 1; in which 61 ATD patients free from rheumatic manifestations were assessed for the presence of these nonorgan-specific autoantibodies against healthy 61 control group, followed by 2nd phase longitudinal clinical follow-up in which cases are monitored systematically to establish occurrence and progression of any rheumatic disease in association to these autoantibodies with its influences and prognosis.

Regarding ATD patients, ANA, anti-dsDNA, Anti-ENA, and RF were present in a percentage of (50.8%), (18%), (21.3%), and (34.4%), respectively, with statistically significance difference (*P* < 0.5) rather than controls. Nearly one third of the studied group (32.8%) developed the rheumatic diseases, over 2 years follow-up. It was obvious that those with positive anti-dsDNA had higher risk (2.45 times) to develop rheumatic diseases than those without. There was a statistically significant positive linear relationship between occurrence of disease in months and (age, anti-dsDNA, anti-CCP, RF, and duration of thyroiditis). Anti-dsDNA and RF are the most significant predictors (*P* < 0.0001).

ATD is more associated with rheumatic diseases than previously thought. Anti-dsDNA, RF, and anti-CCP antibodies may be used as predictive screening markers of systemic lupus erythematosus and RA, with early referral to rheumatologists for close follow-up and early diagnoses for appropriate disease management of the disease, as early disease control will allow better quality of life.

## 1 Introduction

Rheumatic diseases are considered the 2nd greatest cause of disability in the world and have the 4th greatest impact on the overall health.^[1]^ They elicit a major burden on individuals, health, and social care systems, with indirect costs predominance. This overall burden has been recognized by the United Nations and World Health Organization, by endorsing the Bone and Joint Decade 2000 to 2010.^[2]^ The rheumatic disease burden has been increased dramatically by 45% over the last 20 years.^[3,4]^

Hashimoto thyroiditis and Graves disease, which are known as autoimmune thyroid diseases (ATDs), are organ-specific autoimmune disorders characterized by the presence of antibodies against the thyroglobulin, thyroid peroxidase, or thyrotropin receptor autoantigens.^[5]^ Although ATD specific antibodies as antithyroglobulin and antithyroid peroxidase have also been reported in many patients with nonthyroid diseases, and also in healthy population.^[6,7]^

Existence of ATD among patients with systemic autoimmune diseases, such as systemic lupus erythematosus (SLE), rheumatoid arthritis (RA), or Sjögren syndrome (SS), had been well recognized.^[8]^ On the other hand, a high prevalence of other autoantibodies directed against specific nonthyroid antigens has been described in patients with ATD, such as antinuclear antibodies (ANA), antidouble-stranded deoxyribonucleic acid (anti-dsDNA), and antiextractable-nuclear antigens (anti-ENAs) whose clinical meaning is unknown.^[9,10]^

It is well documented that autoimmune diseases may occur years after the presence of its associated autoantibodies.^[11]^ So, autoantibodies may also predict specific diseases and rate of progression.^[12]^ These markers identifications and assessment has predictive value, which might improve secondary prevention of autoimmune disease and help tertiary prevention of disease complications.^[13]^

Our study aimed to assess the prevalence of various nonorgan-specific autoantibodies in patients with ATD, investigate the possible association between these autoantibodies with occurrence of rheumatic diseases and if these autoantibodies might be a predictor parameters for autoimmune rheumatic diseases in those patients.

## 2 Subjects and methods

The study was conducted in 2 main phases, phase 1 was a case–control study followed by a longitudinal clinical follow-up study in which cases were monitored systematically to establish occurrence of any rheumatic diseases.

Sixty one ATD patients with positive antithyroid antibodies were enrolled to our study from Al Hada Armed Forces Hospital, KSA outpatient clinics. The inclusion criteria included males or females of any age fulfilling the criteria of diagnosis of ATD.

The diagnosis of ATD is made according to established criteria based on laboratory markers including thyroid hormone levels (TSH, fT4, fT3), the detection of antithyroid antibodies (antithyroid peroxidase, antithyroglobulin, and antithyrotropin receptor antibodies), and on ultrasound examination (imaging signs of thyroid autoimmunity) of thyroidal gland. The cases were distributed as 2 Gravis disease and 59 Hashimoto thyroiditis. Exclusion criteria included any nonimmune thyroid disease, any patient with sign and symptoms suggestive of connective tissue disease, diagnosed as autoimmune rheumatic disease, or had a history of any other systemic autoimmune disorders, or any other systemic disease.

Sixty-one healthy age and sex matched subjects with mean age of (27.03 ± 14.96) without any thyroid, connective tissue, or autoimmune disease acted as controls.

All subjects underwent full history taking and complete physical examination, blood tests (of complete blood count using Coulter Counter T660, erythrocyte sedimentation rate by Westergren method, C-reactive protein using the enzyme-linked immunosorbent assay technique, urea, creatinine, aspartate amino-transferase, alanine aminotransferase, and albumin), urine tests; all subjects were examined for the presence of rheumatoid factor (RF), and anticyclic-citrullinated peptides (anti-CCP) antibodies done by the chemi-luminescence-micro-particle immunoassay with cut-off 5 for being positive and ANA done by immune florescent assay (IIFA) on HEp-2 in their sera with cut-off of 1:40 for being positive. ANA positive subjects further evaluated by immunological studies for other more specific autoantibodies such as anti-dsDNA by IIFA with cut-off 200 for being positive, as well as antibodies against the extractable nuclear antigens (anti-ENA) (anti-Ro/SSA, anti-La/SSB, anti-uRNP, anti-Sm, anti-centromere, anti-Scl 70, and anti-Jo1 antibodies) using enzyme-linked immunosorbent assay technique.

ATD patients and controls were evaluated for the prevalence of nonorgan-specific antibodies (ANA, anti-ENA, anti-dsDNA, RF, and anti-CCP) with complete history and physical examination for presence of related rheumatologic manifestations at presentation.

The 2nd stage of the study was conducted over 2 years of follow-up period to assess the risk of developing autoimmune diseases related to these autoantibodies among cases with ATD.

During 2 years follow-up of ATD patients, the diagnosis of rheumatic diseases were done according to the following; 2010 RA classification criteria^[14]^ for RA, the International League of Associations for Rheumatology classification of juvenile idiopathic arthritis,^[15]^ the American College of Rheumatology classification criteria of SLE,^[16]^ the American European Consensus Group criteria for primary SS^[17]^ Kahn criteria for mixed connective tissue disease (MCTD),^[18]^ and the International Study Group diagnostic criteria for Behçet disease.^[19]^ Fibromyalgia diagnosed by American College of Rheumatology diagnostic criteria for fibromyalgia 2010.^[20]^

The study was approved by the research and ethical committee at Al Hada Armed Forces Hospital, KSA. Written informed consent from all patients or their guardians had been obtained with explanation of the study purpose and ensuring privacy.

### 2.1 Statistical analysis

Data were coded and statistical analysis was done using Statistical Package for Social Sciences software program (SPSS) Version 20. Quantitative data were expressed as means ± standard deviation. Although qualitative data were expressed as percentages. A Chi-square and Fisher exact tests were applied for bivariate data analysis to test the statistical significance of associations between occurrence of rheumatic diseases and different categorical variables along with the respective relative risk for the occurrence of rheumatic diseases with their 95% confidence intervals, the level of significance was *P* ≤ 0.05.

Multiple regression analysis was done to explore the relationship between 1 continuous dependent variable and a number of independent variables or predictors, how well a set of variables is able to predict a particular outcome and which variables the best predictor of an outcome. Survival curve was constructed by plotting survival rates at different times.

## 3 Results

Our results revealed that 65.6% of patients were adults (>18 years) and 34.4% were children and adolescents (≤18 years). Females constituted 83.6% of the study population. There were various percentages distribution of the different markers positivity with the highest percentage for ANA (50.8%) and RF (34.4%), anti-CCP (19.7%), anti-ENA (21.3%) (anti-Ro/SSA, anti-La/SSB [14.8%], anti-uRNP [8.2%], and anticentromere [1.6%]. None of the patients were positive to anti-Smith, anti-Scl 70, and anti-Jo1). Nearly one third of the studied group (32.8%), developed rheumatic diseases during the follow-up period, distributed as 45% as RA, 10% as SS, 20% as SLE, and only 25% for Behcets, fibromyalgia, MCTD, and Rhupus.

Patients with ATD showed a significant higher prevalence of anti-ENA, ANA, anti-Ro/SSA, anti-La/SSB, anti-dsDNA, RF, and anti-CCP antibodies in comparison with the control group (Table 1). However, there is no significant difference in the prevalence of anti-CCP in association with RF.

**Table 1:**
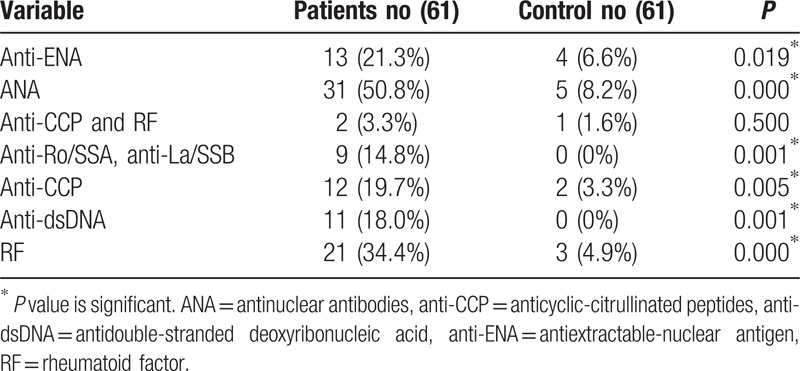
Nonorgan-specific autoantibodies prevalence among patients and control.

**Figure 1:**
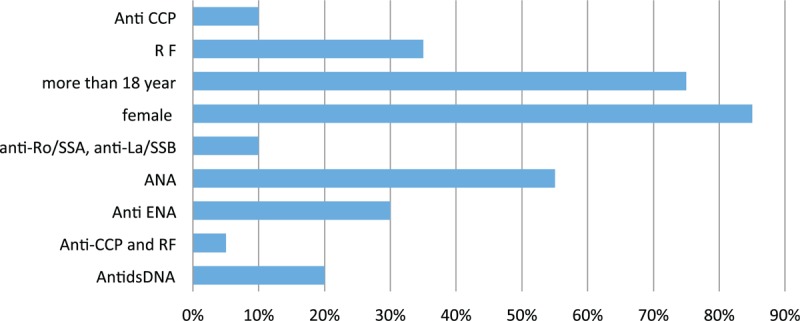
Descriptive statistics of the occurrence of rheumatic diseases regarding different variables.

**Figure 2:**
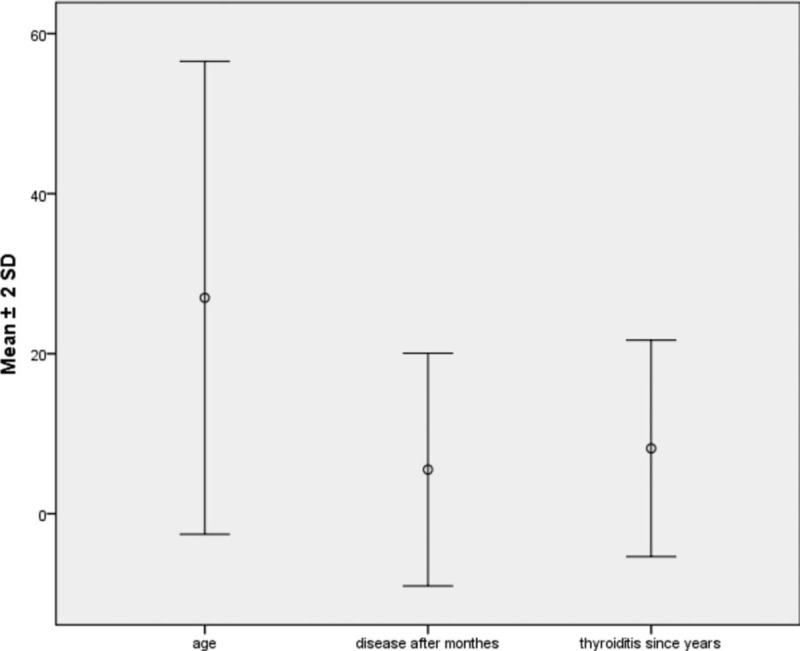
Simple error plot graph with mean values and standard deviation of some quantitative variables.

**Figure 3:**
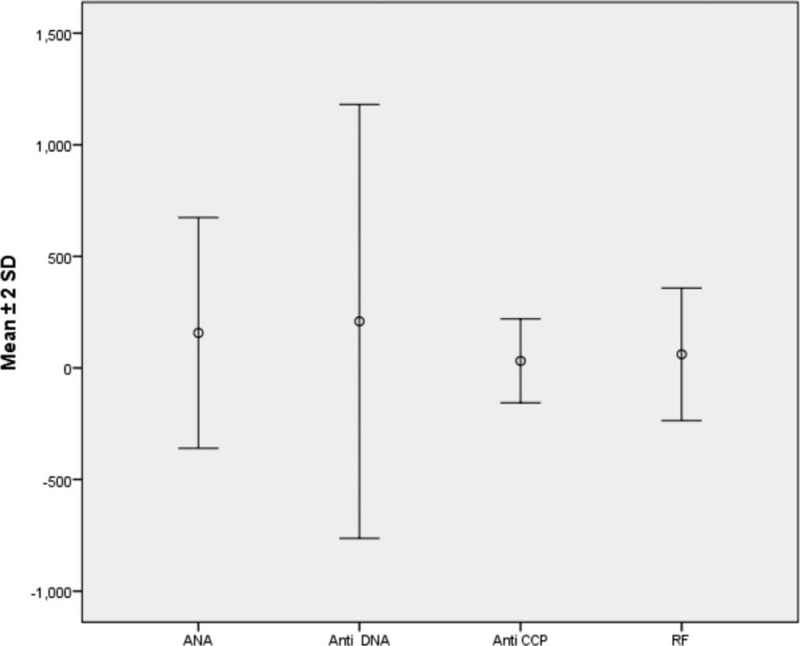
Simple error plot graph with mean values and standard deviation of different markers titers.

**Figure 4:**
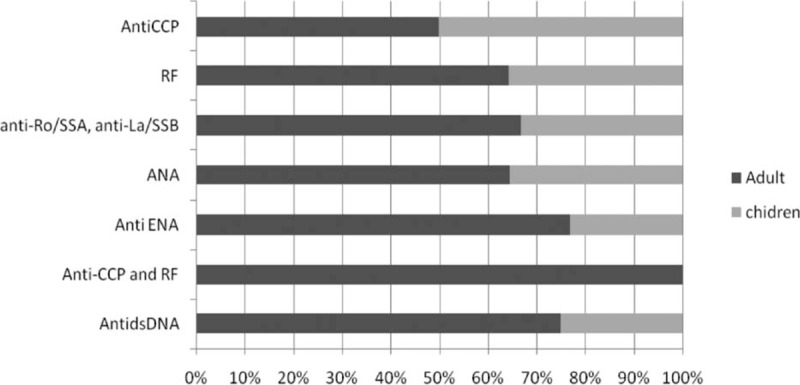
Distribution of different markers positivity regarding age groups.

It was shown in Fig. 1 that those who developed disease were in those who are more than 18 years (75%), mostly females (85%), and shows various percentage distribution of different markers with highest percentage for ANA (55%). The mean age of the studied group was 26.98 ± 14.77, duration of thyroiditis in years was 8.16 ± 6.77, and disease occurrence in months was 5.51 ± 7.27, as shown in Fig. 2. The means of different markers titers are illustrated in Fig. 3. Also there was no statistical significance difference in the distribution of the previous autoantibodies positivity regarding both age groups (*P* > 0.05) (Fig. 4).

The variable anti-dsDNA was significantly associated with the occurrence of disease (*P* < 0.05) (Table 2). The risk of having rheumatic disease for females, with age more than 18 years, and with positive RF, anti-CCP, RF in association with anti-CCP, ANA, anti-dsDNA, anti-ENA, especially anti-uRNP, is more than male, age less than or equal to 18 years, and in association with negative markers, respectively. The highest risk value was found for RF in association with anti-CCP (3.16) and also was found to anti-dsDNA (2.45) times.

**Table 2:**
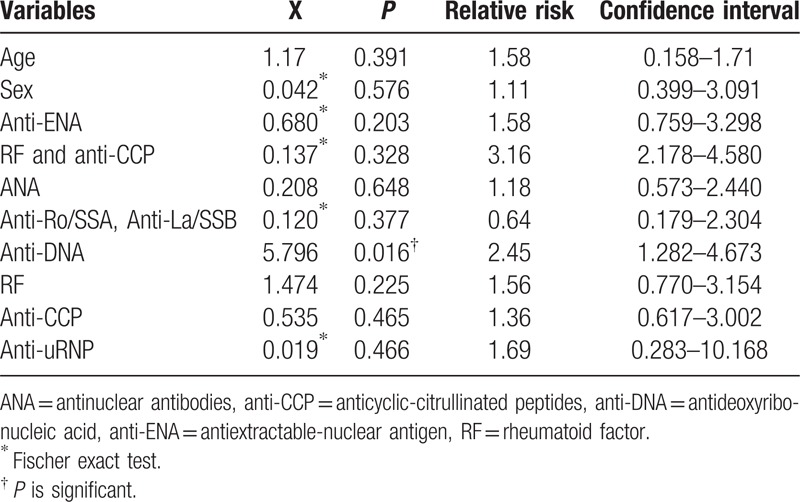
The association between demographic variables and different markers percentages and the occurrence of disease.

There is a statistically significant positive correlation between: age and thyroiditis duration, disease occurrence in months and anti-dsDNA titer, and ANA and anti-dsDNA titer (Table 3).

**Table 3:**
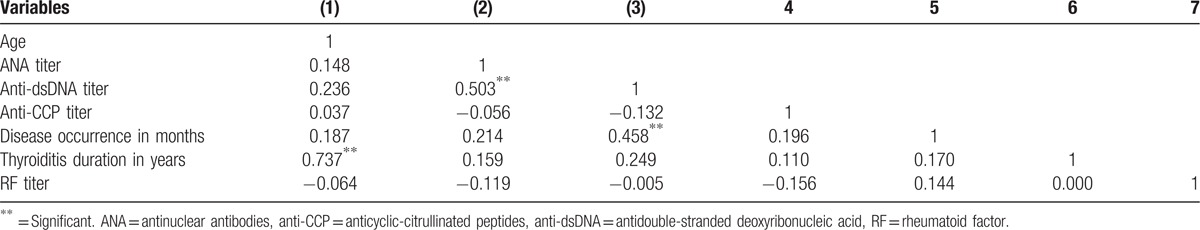
Correlation matrix of different markers titers, mean age, duration of thyroiditis, and time of occurrence of disease.

Table 4 showed a statistical significant positive linear relationship between occurrence of disease in months and these variables (age, anti-dsDNA, anti-CCP, RF, and duration of thyroiditis). Anti-dsDNA and RF are the most significant predictors with the occurrence of disease in months scores as the dependent variable, the model was highly significant (*F*-ratio = 5.75 and *P* < 0.0001). This model accounted for 39% of the total variance in the dependent variable. The main predictors of the occurrence of disease in months were anti-dsDNA and RF.

**Table 4:**
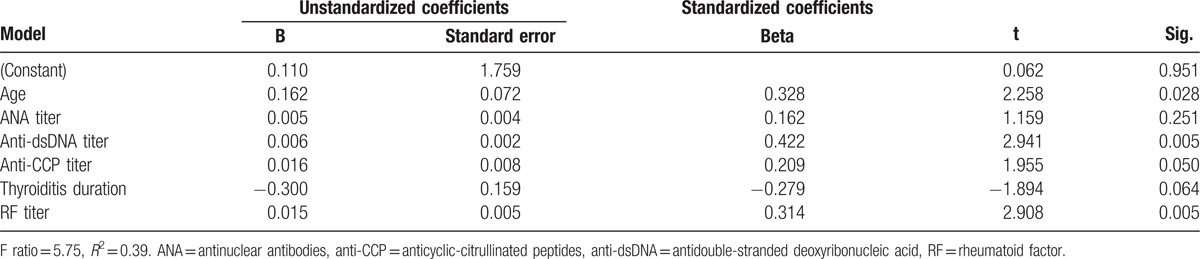
Linear regression between occurrence of disease in months, different markers titers, mean age, and duration of thyroiditis.

The interval start time was divided into 8 intervals with descriptive statistics of each interval regarding disease occurrence. The largest hazard rate (the time of greatest risk) was between months 21 and 23 when the hazard rate goes up to 16% (Table 4).

**Figure 5:**
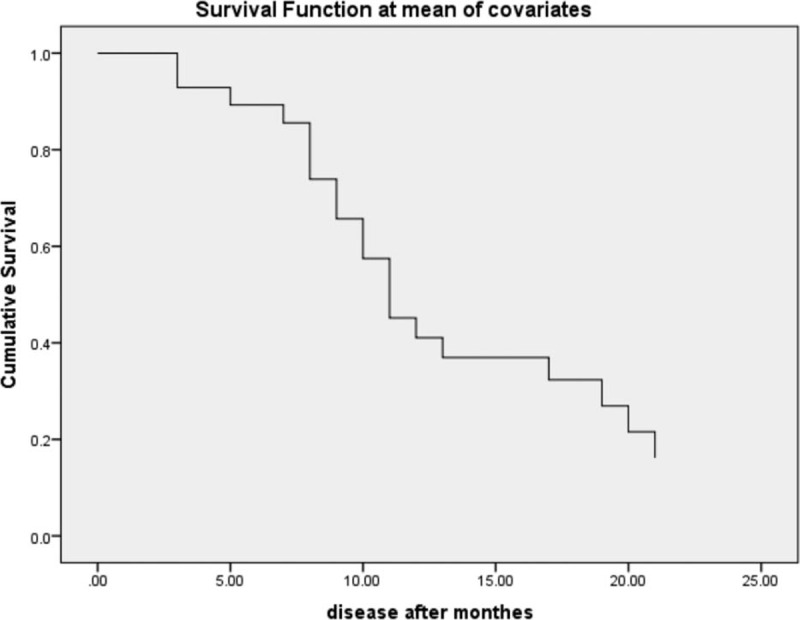
Survival curve of the studied group.

Table 5 demonstrates the time at which 50% of those who originally started out have had the disease regarding different variables. On the other hand, Fig. 5 illustrates the different survival times (free of disease) of the studied group. The analysis was performed with Cox regression model. Table 6 shows the survival curve of the studied group and the cumulative incidence of the disease over months.

**Table 5:**
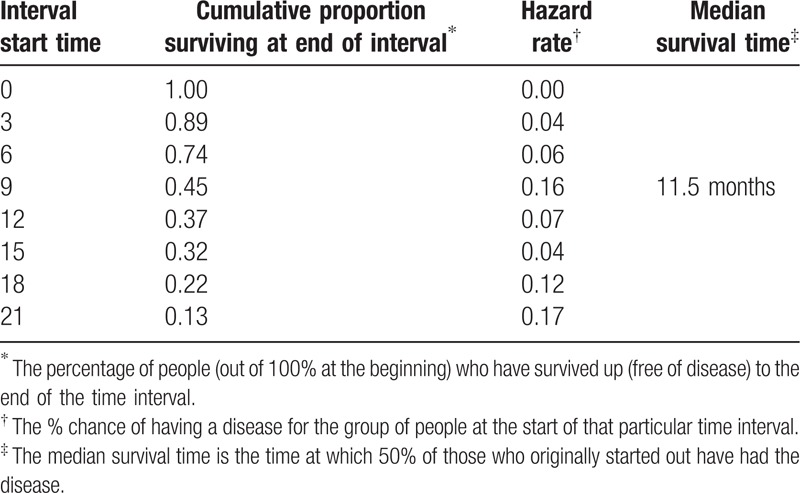
Life tables of the studied group.

**Table 6:**
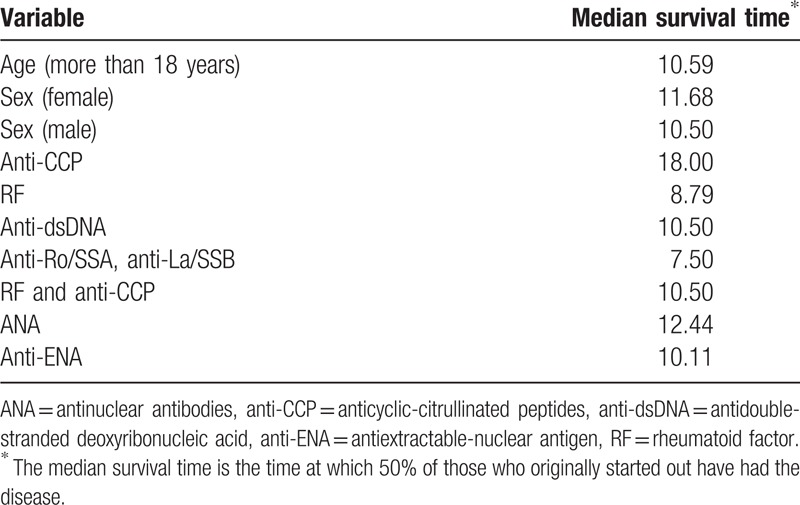
Median survival time in months regarding different variables.

## 4 Discussion

Rheumatic diseases have been described to be associated with autoimmune thyroiditis (ATD), especially hypothyroidism. ATD is commonly associated with nonorgan-specific autoantibodies.^[10]^ Increased prevalence of these autoantibodies in ATD without any evidence of any rheumatic disease is unclear.^[21]^ ANA is considered the most frequent nonorgan-specific antibody in patients with ATD, while other autoantibodies occur rarely.^[22]^ ANA could be detected in variant of autoimmune disorders (i.e., SS, SLE, MCTD, progressive systemic sclerosis, juvenile idiopathic arthritis, and autoimmune hepatitis) as well as in some infectious diseases,^[23,24]^ and also found in healthy populations.^[24]^ High prevalence of ANA among ATD patients had been well recognized, it has been reported prevalence up to 45% in ATD adults,^[9,22]^ and was 13.8% in a recent cross-sectional study older than 12 years.^[25]^

In our study, we used a cut-off of 1:40 for ANA IIFA on HEp-2 cells as well as previous studies, and we found that ANA prevalence was (50.8%) IIFA on HEp-2 cells, is considered the most reliable method for ANA screening.^[23,26]^The presence of other nonthyroid autoantibodies in ATD patients remains controversial. In Tektonidou et al study^[9]^ 12% had anti-dsDNA antibodies,^[27]^ but was much higher 74.5% in Pedro et al study.^[28]^ But in our study anti-dsDNA antibodies were positive in (18%) of ATD patients.

Some studies reported that other nonorgan-specific antibodies were not significantly different among patients with ATD and healthy control,^[21]^ although our study reveals some significant results regarding different antibodies between cases and control.

Serum antibodies against the Ro/SSA and La/SSB antigens have been only sporadically described in ATD.^[9]^ In 1 cross-sectional study about 10% had anti-Ro antibodies, 1 had anti-Ro and anti-La antibodies 0.5%.^[9]^ One of the most comprehensive study including 50 patients with ATD, 13 (26%) had ANA and 17 (34%) had antisingle-stranded DNA antibodies; however, no patient had antibodies against dsDNA, ENAs (anti-Ro/SSA, anti-La/SSB, anti-Sm, and anti-RNP), or RF,^[29]^ which was different than our study, we reported higher percentage regarding anti-Ro/SSA and La/SSB (14.8%), anti-uRNP (8.2%), anti-dsDNA (18.0%), anti-CCP (19.7%), and RF(34.4%).

It is well known now that autoimmune diseases occur many years after the presence of its end-organ autoantibodies.^[11]^ To implicate this concept we conduct the 2nd stage in which we followed our patients over 2 years for the development of any rheumatic manifestations related to these autoantibodies.

Up to our knowledge our prospective study is the 1st to detect the risk of nonorgan-specific autoantibodies in ATD for the development of autoimmune rheumatic diseases. During the 2 years follow-up, nearly one third of ATD patients developed autoimmune disease (32.8%). Many risk factors have been detected to be associated with autoimmune diseases as female gender especially middle age,^[11]^ as it was shown in our study, most of the patients who develops autoimmune diseases were female gender (85%) and mean age was 26.98 ± 14.77.

Up to 6.5% of our patients with ATD and positive ANA developed SS which was in agreement with Tektonidou and his group who had 9% of their patients with ATD and positive for ANA were later diagnosed as primary SS, reinforcing once more the possibility of polyclonal autoimmune response to organ-specific and nonspecific autoantigens.^[9]^

In a retrospective study, Sera of 130 SLE patients were obtained before SLE diagnosis. The autoantibodies tested were present in at least 88%, a mean of 3.3 years before the diagnosis. ANA, anti-Ro/SSA, anti-La/SSB, and anti-phospholipid antibodies first appeared prior to the diagnosis of SLE with a mean of 3.4 years and a mean of 2.2 in anti-dsDNA, making them the one of the important disease predictors.^[30]^

Our study's comparable results showed that patients of ATD and positive anti-dsDNA have 2.45 times risk to develop rheumatic disease, and all of our ATD patients who developed SLE were positive for anti-dsDNA, within the 2 years follow-up.

In a large cohort study carried by Boelaert et al,^[27]^ the most prevalent coexisting autoimmune disease in subjects with ATD was RA, which was concomitant with our findings of RA in 45% of patients. That was in contrast to Tektonidou study where SS was the highest.^[9]^

Autoantibodies in RA may be present before clinical disease is well known.^[31]^ Recently anti-CCP antibodies are present in the blood years before disease onset; patients were shown to have positive RF and/or anti-CCP on at least once before RA symptoms, a median of 4.5 years before symptom onset.^[31]^ In another similar study by Rantapää-Dahlqvist et al,^[32]^ the prevalence of autoantibodies were 16.9% for IgG-RF, 19.3% for IgM-RF, and 33.7% for anti-CCP, with median 2.5 years before RA. The risk of developing autoimmune diseases was maximum at the end of our study. In our study, the ATD patients with positive RF in association with anti-CCP showed higher risk 3.16 times to develop RA than those without.

This study showed a statistically significant positive linear relationship between age, anti-dsDNA, anti-CCP, RF, and duration of thyroiditis with the occurrence of rheumatic diseases, making anti-dsDNA and RF are the most significant predictors.

General population screening; to identify high-risk individuals of some diseases is an essential real concept and strategy. The goal of early identification could be either prevention or limitation of clinical disease impact.^[15]^ The high-risk groups testing may change the autoantibody serological tests positive predictive value.^[33]^

It was obvious in our study that ATD is more associated with rheumatic diseases than previously thought. So, early referral to rheumatologists is needed to differentiate the extra glandular ATD manifestations from true associated autoimmune disease. Anti-dsDNA, RF, in association with anti-CCP antibodies may be used as predictive markers of SLE and RA, respectively, in ATD patients for close follow-up and early diagnoses with appropriate disease management of the disease. However, more large-cohort longitudinal studies with stratification of the studied group to adults and children are needed to establish this concept in routine serological testing for ATD patients, as identification of autoimmune diseases in the preclinical stage allow the possibly of disease control and better quality of life.
